# Supplementary figures and images for: Perceived Benefits of Nature in Diverse Populations
Source: Int J Environ Res Public Health. 2025 Apr 4;22(4):563. doi: 10.3390/ijerph22040563 (PMC12026635; doi:10.3390/ijerph22040563)

**Supplementary Figure S1: Study Flow Chart**

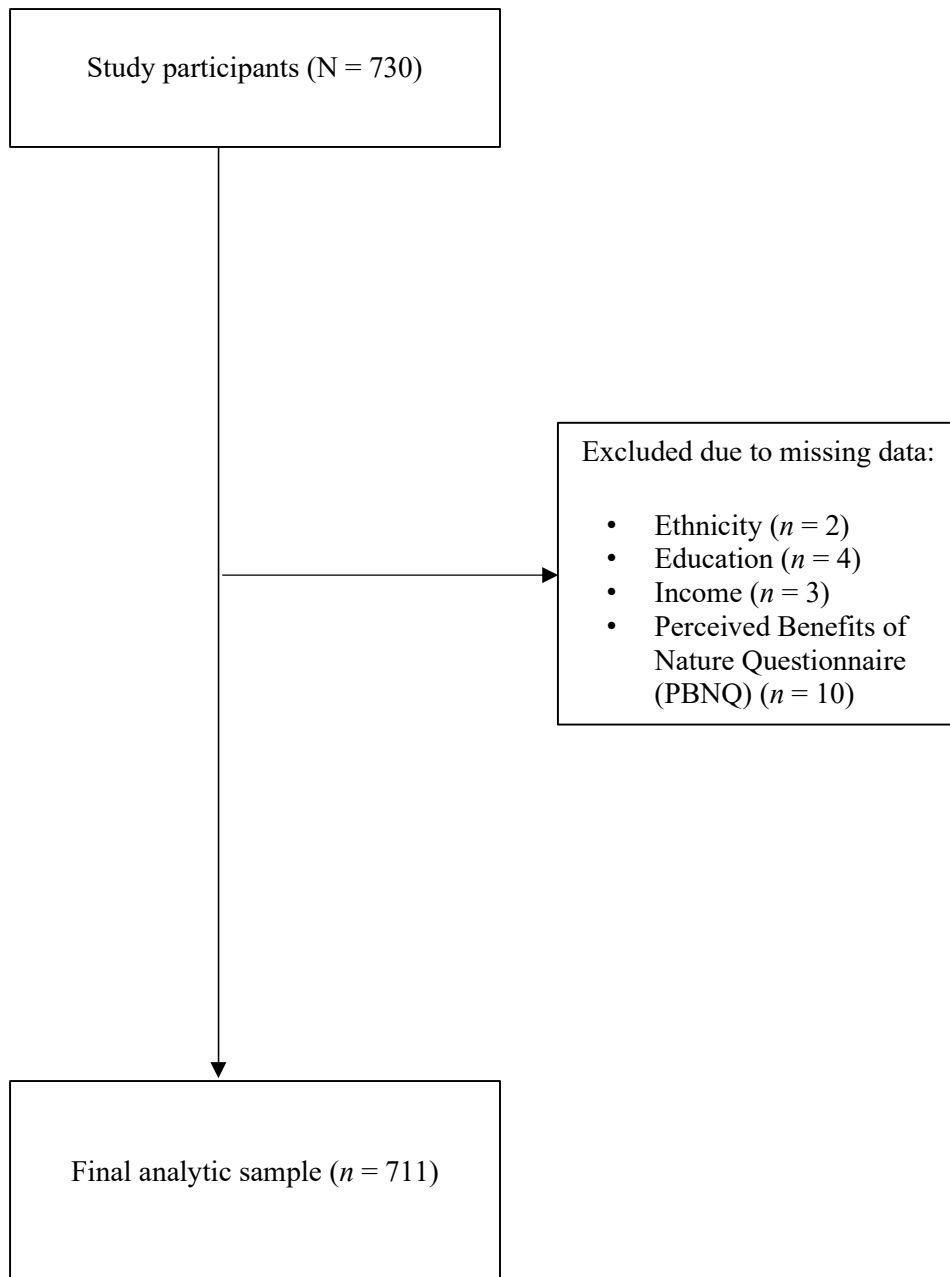

Supplement: Supplementary file 1 [file ijerph-22-00563-s001.zip › ijerph-3478254-supplementary.pdf]
